# Cost-effectiveness of rotavirus vaccination in the Philippines: A modeling study

**DOI:** 10.1016/j.vaccine.2021.09.075

**Published:** 2021-11-26

**Authors:** Maria Esterlita T. Villanueva-Uy, Hilton Y. Lam, Josephine G. Aldaba, Tristan Marvin Z. Uy, Haidee A. Valverde, Maria Wilda T. Silva, Jessica Mooney, Andrew Clark, Clint Pecenka

**Affiliations:** a Institute of Child Health and Human Development, University of the Philippines Manila-National Institutes of Health, Manila, Philippines; b Institute of Health Policy and Development Studies, University of the Philippines Manila-National Institutes of Health, Manila, Philippines; c Disease Prevention and Control Bureau, Department of Health, Manila, Philippines; d Center for Vaccine Innovation and Access, PATH, Seattle, WA, USA; e Department of Health Services Research and Policy, London School of Hygiene and Tropical Medicine, UK

**Keywords:** Rotavirus, Vaccination, Cost-effectiveness, Philippines

## Abstract

**Introduction:**

Rotavirus gastroenteritis (RVGE) remains a leading cause of hospitalization and death in children under five years of age in the Philippines. Rotavirus (RV) vaccination was introduced into the national immunization program (NIP) in 2012 but has since been limited to one region due to cost considerations and conflicting local cost-effectiveness estimates. Updated estimates of the cost-effectiveness of RV vaccination are required to inform prioritization of national immunization activities.

**Methods:**

We calculated the potential costs and benefits of rotavirus vaccination over a 10-year-period (2021–2031) from a government and societal perspective, comparing four alternative rotavirus vaccines: Rotavac, Rotasiil, Rotarix and Rotateq. For each vaccine, a proportionate outcomes model was used to calculate the expected number of disease events, DALYs, vaccination program costs, and healthcare costs, with and without vaccination. The primary outcome measure was the cost per DALY averted. Assuming each product would generate similar benefits, the dominant (lowest cost) product was identified. We then calculated the cost-effectiveness (US$ per Disability Adjusted Life Year [DALY] averted) of the least costly product and compared it to willingness-to-pay thresholds of 0.5 and 1 times the national GDP per capita ($3,485), and ran deterministic and probabilistic sensitivity analyses.

**Results:**

Introducing any of the four rotavirus vaccines would avert around 40% of RVGE visits, hospitalizations, and deaths over the period 2021–2031. Over the same ten-year period, the incremental cost of vaccination from a government perspective was estimated to be around $104, $105, $220, and $277 million for Rotavac, Rotasiil, Rotarix and Rotateq, respectively. The equivalent cost from a societal perspective was $58, $60, $178 and $231 million. The cost-effectiveness of the least costly product (Rotavac) was $1,148 ($830–$1682) from a government perspective and $646 ($233–1277) from a societal perspective. All other products offered similar benefits but at a higher cost. There is a >99% probability that Rotavac would be cost-effective at a willingness-to-pay threshold set at 0.5 times the national GDP per capita.

**Conclusion:**

Both Rotavac and Rotasiil are likely to be cost-effective options in the Philippines, but it is not possible to say definitively which product should be preferred. Rotarix and Rotateq are expected to offer similar benefits at more cost, so would need to be priced far more competitively to be considered for introduction.

## 1 Introduction

Globally, diarrhea causes over 500,000 deaths in children less than five years old annually, comprising almost 10% of childhood mortality [1]. Although diarrheal deaths have been decreasing worldwide, acute watery diarrhea has remained a leading cause of under-five mortality in the Philippines [1], [2]. In 2018 alone, it accounted for 6.7 deaths per 100,000 under-five children nationwide [2].

Rotavirus (RV) has been a major etiology of diarrhea. It has comprised approximately 44% of diarrheal hospitalizations and 28% of deaths globally [3]. In the Philippines, the RV-positivity rate among children hospitalized with diarrhea has ranged from 30 to 53% [4], [5], [6], [7], [8]. It is estimated that 3.7% of all-cause under-five deaths in the country are due to rotavirus diarrhea [7], [8]. Accordingly, in 2009, the WHO recommended the inclusion of RV vaccination into national immunization programs (NIPs) [9], and Rotarix was introduced in the Philippine NIP in 2012. However, vaccine distribution was limited to a single region in 2014 due to operational issues in targeting infants belonging to households in the poorest quintile [10]. To date, there has been no scale-up of the program due to cost considerations and conflicting local cost-effectiveness estimates.

A number of studies have shown RV vaccination to be cost-effective in low- and middle-income countries (LMIC), including non-Gavi countries [11], [12], [13]. Cost-effectiveness analyses (CEAs) in the Philippines have reported conflicting results. In 2014, Lee et al. found Rotarix to be cost-effective (cost per Quality Adjusted Life Year gained of 12% of the national GDP per capita) based on a static model assuming a price per fully immunized child (FIC) of around $20 [14]. In 2018, these results were supported by Dizon et al. using updated data [15]. In 2015, Lam et al. found that neither Rotarix nor Rotateq would be cost-effective (cost per DALY averted of around four times the national GDP per capita) based on a dynamic transmission model assuming a price per FIC of around $20. As such, the authors concluded both vaccines would need be priced below $6 per FIC to generate a cost per DALY averted below the national GDP per capita [16].

Newer RV vaccines with different price points have subsequently been pre-qualified by the WHO [17], although Rotavac and Rotasiil have yet to enter the local market. Furthermore, with the passage of Universal Healthcare Act of 2019, the Philippines has institutionalized the Health Technology Assessment (HTA) as a mechanism integrating CEAs in the determination of services, including vaccines, to recommend to two governmental bodies: the Department of Health (DOH) and the Philippine Health Insurance Corporation (PhilHealth) [18]. These developments underscore the need for a local re-evaluation of RV vaccination to guide decision-makers on its expansion in the NIP. In this study, we aim to determine the cost-effectiveness and financial implications of an RV vaccination program over a 10-year period (2021–2031) in the Philippines.

## 2 Methods

### 2.1 Study design

A universal vaccine decision-support model (UNIVAC version 1.4.13) was used to calculate the potential cost-effectiveness and health impact of rotavirus vaccination over a 10-year period (2021–2031) in the Philippines. This static Microsoft Excel-based proportionate outcomes model was developed specifically for use by national multidisciplinary teams in LMICs [19]. Our primary outcome measure was the cost (in USD) per DALY averted of rotavirus vaccination, from government and societal perspectives, compared to no vaccination. We compared four WHO pre-qualified RV vaccines (Rotavac, Rotasiil, Rotarix, and Rotateq) to no vaccination, and to each other. We also compared the cost-effectiveness of the RV vaccines to the willingness-to-pay thresholds of 0.5 and 1 times the national Gross Domestic Product (GDP) per capita ($3,485 USD).

To populate the model, we carried out a review of international and local literature, government records, and the PhilHealth claims database. Local data, especially primary data from local health agencies, were prioritized when available. A local expert panel comprised of 15 multidisciplinary specialists (e.g., pediatric infectious disease specialists, pediatric gastroenterologists, vaccinologists, pediatric surgeons, maternal and child nurses, health economists, public health practitioners, and national and international program managers) were invited to two workshops held on December 9, 2020 and March 24, 2021 to review the accuracy, completeness, and appropriateness of the data inputs (e.g., disease burden, vaccine coverage and timeliness, vaccine safety, vaccine efficacy, vaccine costs, healthcare costs), prior to analysis. All demographic projections (population size and life-expectancy by single year of age and single calendar year) were based on the UNPOP 2019 revision projections for the Philippines [20].

The potential benefits of RV vaccination were projected by estimating the number of RVGE disease cases, visits, hospitalizations, deaths, and Disability-Adjusted Life Years (DALYs), with and without vaccination, among children less than five years of age.

‘Cases’ refer to children in the community with RVGE, regardless if brought to a healthcare facility. ‘Visits’ constitute the cases brought (but not admitted) to a healthcare facility (e.g., outpatient clinic or emergency room). Meanwhile ‘hospitalizations’ are defined as cases admitted to an inpatient healthcare facility. RVGE was further classified as non-severe or severe. Non-severe RVGE was presumed to always lead to recovery. Thus, associated events include only cases (non-severe) and visits (outpatient clinic). In contrast, severe RVGE cases involve visits (emergency room), hospitalizations, and deaths.

We used previously described methods to calculate the potential number of excess cases, hospitalizations, and deaths associated with intussusception, a possible serious adverse event following RV vaccination [21].

We estimated healthcare treatment costs with and without vaccination. We included costs associated with non-severe RVGE visits, severe RVGE visits and hospitalizations, and hospitalizations from intussusception. We calculated healthcare costs from two perspectives: 1.) the government perspective, representing all costs borne by the payor (i.e., the national government), and 2.) the societal perspective, pertaining to all incurred direct and indirect costs irrespective of payor.

We conducted deterministic sensitivity analyses (DSA) using discount rates of 3%, 5%, and 10% (7% as the base case according to HTA guidelines) [22], and ran scenarios using the high and low estimates of: disease event rates, vaccine coverage rates, vaccine efficacy and safety, vaccine price, health system cost projection, and healthcare costs. We also performed probabilistic sensitivity analyses (PSA) on the same parameters and ran the simulation 1,000 times to create a cost-effectiveness acceptability curve to ascertain the probability of cost-effectiveness of the four RV vaccines at willingness-to-pay thresholds of 0.5 and 1 times the GDP per capita ($3,485).

### 2.2 Model parameters

#### 2.2.1 Disease burden

A summary of the model inputs can be found in Table 1.

**Table 1:** Data inputs for estimating the burden of rotavirus gastroenteritis and intussusception before the introduction of rotavirus vaccination.^a^

Model Parameter	Point Estimate (Uncertainty Range)	Sources
Non-severe RVGE^b^		
Cases	1,798 (1,348–2,248)	[6], [23]
Visits	755 (566–944)	[6]
Severe RVGE^b^		
Cases	813 (610–1,016)	[6], expert opinion
Visits	451 (338–564)	[6]
Hospitalizations	281 (211–389)	[5], [6]
Deaths	19 (13–28)	[21]
Intussusception^b^		
Cases	59.56 (5.52 – 225.40)	[23], [32]
Hospitalizations	51.82 (4.80–196.10)	[32]
Deaths	6.99 (0.65–26.61)	
Percentage of healthy time lost^c^		
Non-severe RVGE	18.8 (12.5–26.4)	[27]
Severe RVGE	24.7 (16.4–34.8)	
Intussusception	32.4 (22.0–44.2)	
Duration of illness^d^		
Non-severe RVGE	0.01 (0.01–0.02)	[31], expert opinion
Severe RVGE	0.02 (0.01–0.03)	[5], [6], [28], [29], [30], expert opinion
Intussusception	0.02 (0.003–0.027)	[28], [29], [30], expert opinion

a All parameters assumed to have Beta-PERT distribution.

b Estimates in annual age-specific rates of per 100,000.

c Estimates in percent.

d Estimates in years.

##### 2.2.1.1 RVGE

Our literature search yielded several local incidence studies with a wide range of RV positivity rates (30–40%) [4], [5], [6]. Therefore, we based our estimates on the most recent cross-sectional study by Carlos et al., which reported a positivity rate of 15% for outpatient visits and ∼30% for ER visits/hospitalizations [6]. Because the facility-based study involved only visits and hospitalizations, the number of cases was derived using health-seeking rates reported elsewhere. For non-severe RVGE, we assumed 42% of cases would be brought to a healthcare facility, as reported in the 2017 National Demographic Health Survey [23]. For severe RVGE, we applied a higher health-seeking rate (90%) [24], based on our local expert panel and international data showing rates differed by diarrheal severity [25], [26]. We produced high and low estimates of disease burden by adjusting the base case by ±25%. However, for hospitalizations, we used the upper bound estimate based on the highest RV positivity rate (42.9%) found among the local studies [5]. We did not find local data on RVGE deaths. Instead, we used estimated death rates from a modelling study by Clark et al. based on pooled data from three sources of rotavirus mortality estimates for the Philippines [21].

To estimate DALYs, we obtained disability weights for moderate and severe diarrhea from the Global Burden of Disease study (GBD) [27]. For severe RVGE, duration of illness was a composite of: 1.) average length of hospital stay based on insurance claims [28], [29], [30]; 2.) average pre-hospital symptomatic period reported by Santos et al. [5] and 3.) opinion from our expert panel. Claims data were consistent with findings from Carlos et al. [6]. Meanwhile, other local studies reported a shorter duration, which we used as a lower estimate. The upper estimate corresponded to the base case plus the standard deviation from Santos et al. [5]. In contrast, for duration of non-severe RVGE, we found no local data and acquired the estimates from the Centers for Disease Control and Prevention and from our local expert panel [31].

##### 2.2.1.2 Intussusception

We found no data on local incidence and mortality rates of intussusception. For hospitalization and deaths, we used region-specific estimates from a global systematic review and meta-analysis [32]. To estimate the incidence of intussusception cases, we used the hospitalization rate, and inflated it to account for the small proportion of infants without access to hospitals (using diphtheria-tetanus-pertussis (DTP) dose 1 vaccine coverage as proxy for healthcare access [23]). Disability weights for intussusception were taken from GBD (using severe abdominopelvic problem as a proxy) [27]. For duration of illness, the point estimate was computed from PhilHealth data while the range was provided by the local expert panel [28], [29], [30].

#### 2.2.2 Vaccine coverage, timeliness, efficacy, and safety

A summary of the model inputs can be found in Table 2.

**Table 2:** Data input for estimating vaccine coverage rates, timeliness, efficacy, and safety.^a^

Model Parameter			Point Estimate (Uncertainty Range)	Sources
Vaccine coverage rates^b^				
Dose 1			71.60 (63.3; 81.2)	[33], [34], [35], [36], [37]
Dose 2			70.5 (57.0; 86.0)	
Dose 3			69.2 (55.3; 85.0)	
Timeliness (coverage by age)^c^				
Dose 1		4 weeks	2%	[33], [34], [35], [36], [37], [38]
		8 weeks	40%	
		12 weeks	60%	
		24 weeks	70%	
		1 year	71%	
Dose 2		4 weeks	0%	
		8 weeks	0.1%	
		12 weeks	28%	
		24 weeks	66%	
		1 year	71%	
Dose 3		4 weeks	0%	
		8 weeks	0%	
		12 weeks	0%	
		24 weeks	53%	
		1 year	68%	
		>1 year	69%	
Vaccine efficacy^d^				
Rotarix		Dose 1	48% (−138% − 89%)	[10]
		Dose 2	67% (2% − 89%)	
Rotateq, Rotavac, Rotasiil		Dose 1	48% (−138% − 89%)	
		Dose 2	67% (2% − 89%)	
		Dose 3	67% (2% − 89%)	Assumption
Vaccine Safety^e^				
Rotarix	1–7 days	Dose 1	4.15 (2.27–7.61)	[21]
	1–7 days	Dose 2	1.76 (1.39–2.22)	
	8–21 days	Dose 1	1.51 (1.06–2.16)	
	8–21 days	Dose 2	1.27 (1.0–1.62)	
Rotavac, Rotasiil, RotaTeq,	1–7 days	Dose 1	4.15 (2.27–7.61)	
	1–7 days	Dose 2	1.76 (1.39–2.22)	
	1–7 days	Dose 3	1.00 (1.00–1.00)	
	8–21 days	Dose 1	1.51 (1.06–2.16)	
	8–21 days	Dose 2	1.27 (1.0–1.62)	
	8–21 days	Dose 3	1.00 (1.00–1.00)	

a All parameters assumed to have Beta-PERT distribution.

b Estimates in percent annual coverage.

c Estimates are coverage rates by age in cumulative percentage.

d Model input adjusted to reflect vaccine efficacy two weeks after vaccination; a gamma curve was fitted to data points to determine expected efficacy at different follow-up points.

e Estimates in relative risk ratios.

To account for potential vaccine non-uptake and delay, we used local data on DTP vaccination coverage rates as a proxy for the expected RV vaccine coverage and timeliness. DTP vaccination rates of 72%, 71% and 69% for doses 1, 2 and 3, respectively, were the average annual coverage rates from 2014 to 2018 found in the Field Health Services Information System (FHSIS) [33], [34], [35], [36], [37]. The low and high values were the lowest and highest rates in the set [33], [34], [35], [36], [37]. Timeliness of vaccination was derived from a local records review study from January 2016 to September 2017 by Raguindin et al. [38].

In the Philippines, a case control study by Lopez et al. evaluated the effectiveness of Rotarix against RV positive hospitalizations in children aged <5 years [10]. The two-dose VE was estimated to be 86% among children aged 8–11 months and 67% among those aged 12–23 months. Equivalent estimates for one-dose VE were 74% and 48%, respectively. We used previously described methods [39] to determine the expected VE at different follow-up times after administration of each dose by fitting gamma curves to the available data points. There was insufficient evidence to allow us to differentiate VE by product, so we assumed the same VE for all RV vaccine brands [40], [41]. Since Rotarix only requires two doses while the other types required three, we assumed the same second- and third-dose efficacy for the three-dose RV vaccines.

Our estimates of the relative risk of intussusception in the 1-7- and 8-21-day periods after the first and second doses were based on a global meta-analysis of self-controlled case series (SCCS) studies [21].

#### 2.2.3 Vaccination program costs

A summary of the model inputs can be found in Table 3.

**Table 3:** Data inputs for estimating vaccination program costs and healthcare costs.^a^

Model Parameter	Point Estimate (Uncertainty Range)	Sources
*Vaccination Program Costs*		
Vaccine Price Per Dose (in USD)		
Rotavac	1.06^b^ (0.79–1.32)	[42]
Rotasiil	1.09^b^ (0.82–1.37)	[42]
Rotarix	7.20^c^ (5.40–9.00)	DOH communication
Rotateq	5.57^b^ (4.18–6.97)	[42]
Percentage wastage (%)		
Rotavac	10 (5–15)	[43]
Rotasiil	10 (5–15)	
Rotarix	5 (2–10)	
Rotateq	5 (2–10)	
Safety box	5 (2–10)	
Other charges		
Safety box^d^	0.15 (0.11–0.19)	DOH communication
Local delivery, storage, and handling^e^	6.0 (5.0–7.0)	
Incremental health system cost per dose (in USD)^c^	2.63 (1.97–3.29)	[44]
*Healthcare Cost*^c^, ^d^		
Non-severe RVGE visits		
Government perspective	24.81 (15.51–34.10)	See Supplementary Tables
Societal perspective	39.53 (25.63; 61.64)	
Severe RVGE visits		
Government perspective	65.59 (38.24–84.89)	See Supplementary Tables
Societal perspective	152.68 (90.81–246.40)	
Severe RVGE hospitalizations		
Government perspective	120.92 (90.69–151.15)	See Supplementary Tables
Societal perspective	355.18 (169.98–595.64)	
Intussusception hospitalizations		
Government perspective	203.55 (152.66–254.43)	See Supplementary Tables
Societal perspective	575.71 (358.22–829.40)	

a All parameters assumed to have Beta-PERT distribution.

b Adjusted for inflation.

c 2018 procurement price; estimate originally in PHP adjusted for inflation and then converted using the 2020 average exchange rate (1 USD = 49.62 PHP).

d Estimates converted from PHP using 2020 average exchange rate (1 USD-49.62).

e Estimates in percent of vaccine price.

The government procurement price of Rotarix from 2018 was available from communication with the DOH and used as base case after adjusting for inflation to the 2020 price. The price estimates of the other RV vaccine types were accessed from the Market Information for Access to Vaccines (MI4A) database of the WHO [42]. The estimate for Rotateq was the average of inflation-adjusted prices from non-Gavi-eligible LMICs from 2014 to 2019 while for Rotavac and Rotasiil, the estimates were the averages of inflation-adjusted procurement prices of Gavi-eligible LMICs from 2016 to 2019. The high and low values for all four RV vaccines were ±25% of the base price. The DOH supplied data on two other program expenses: safety box price, which was adjusted by 25% for high and low estimate; and local handling and delivery costs, which were given as a percentage of the vaccine price. We did not find data on local wastage rates; thus, we used the WHO calculator for percentage wastage of all vaccine types and supplies [43]. Rotavac, as a five-dose vial, and Rotasiil, as a two-dose vial, had higher wastage rates than Rotarix and Rotateq, both one-dose vials. Similarly, in the absence of local figures on incremental health system costs, we used the data from the Immunization Delivery Cost Catalogue (IDCC) for LMICs [44] and assumed the same incremental costs for the four RV vaccines.

#### 2.2.4 Healthcare costs

A summary of the model inputs can be found in Table 3.

Detailed costing can be found in Supplementary Tables (Tables 1–4). Briefly, costs were classified into direct and indirect costs. Direct costs, whether medical or non-medical, included the costs of goods and services used in the course of treatment while indirect costs referred to the caregivers’ loss of productivity or income. Direct non-medical costs included transportation costs and meal costs for patients and caregivers while seeking treatment. The government perspective specifically involved only direct medical costs borne by the government as payor; the societal perspective entailed all direct and indirect costs, irrespective of payor.

Costs of healthcare visits for RVGE were adapted from the costing scheme implemented by Lee et al. but substituted with contemporary (2020) prices [14]. Estimated costs of hospitalization for RVGE or intussusception were derived from the PhilHealth database [28], [29], [30]. The average of reimbursed claims were used in the government perspective, while the average of total hospital bills (reimbursed claims plus out-of-pocket expenses) comprise the cost calculations for the societal perspective. For both visits and hospitalizations, we itemized the costs where applicable. We then constructed interval estimates for each item, using data from local literature, the official database, or the local expert panel.

## 3 Results

### 3.1 Impact on healthcare costs and disease events

A summary of healthcare costs and disease events with and without vaccination can be found in Table 4.

**Table 4:** Disease events, healthcare costs, and vaccine program costs over a 10-year period of RV vaccination program.^a^, ^b^, ^c^, ^d^

Parameter	Scenario				
	No vaccination	Rotavac	Rotasiil	Rotarix	Rotateq
**Disease events**					
Non-severe RVGE cases	1,961,554	1,109,332	1,109,332	1,160,267	1,109,332
Severe RVGE cases	886,954	433,603	433,603	460,698	433,603
Intussusception cases	64,978	65,628	65,628	65,628	65,628
Non-severe RVGE visits	823,678	465,821	465,821	487,209	465,821
Severe RVGE visits	492,025	240,535	240,535	255,566	240,535
Severe RVGE hospitalizations	306,561	149,868	149,868	159,233	149,868
Intussusception hospitalizations	56,532	57,098	57,098	57,098	57,098
Severe RVGE deaths	16,794	8,210	8,210	8,723	8,210
Intussusception deaths	6,182	6,244	6,244	6,244	6,244
DALYs	239,190	148,941	148,941	154,294	148,941

**Averted disease events**					
Non-severe RVGE cases	---	852,222	852,222	801,287	852,222
Severe RVGE cases	---	453,351	453,351	426,265	453,351
Intussusception cases	---	−650	−650	−650	−650
Non-severe RVGE visits	---	357,857	357,857	336,469	357,857
Severe RVGE visits	---	251,490	251,490	236,459	251,490
Severe RVGE hospitalizations	---	156,693	156,693	147,328	156,693
Intussusception hospitalizations	---	−566	−566	−566	−566
Severe RVGE deaths	---	8,584	8,584	8,071	8,584
Intussusception deaths	---	−62	−62	−62	−62
DALYs averted	---	90,249	90,249	84,896	90,249

**Healthcare costs**					
Government perspective	71,259,669	39,761,532	39,761,532	41,621428	39,761,532
Societal perspective	175,297,051	98,436,203	98,436,103	102,976,542	98,436,103

**Averted healthcare costs**					
Government perspective	–	31,498,137	31,498,137	29,638,241	31,498,137
Societal perspective	–	76,860,949	76,860,949	72,320,509	76,860,949

**Vaccination program costs**					
Total program costs	---	135,130,458	136,361,521	249,851,158	308,170,403
Vaccine costs	–	43,497,560	44,728,623	188,230,002	216,537,505
Health system costs	–	91,632,898	91,632,898	61,621,156	91,632,898

**Incremental net costs**^e^					
Government perspective	---	103,632,321	104,863,384	220,212,917	276,672,266
Societal perspective	---	58,269,509	59,500,572	177,530,649	231,309,454

a All parameters assumed to have Beta-PERT distribution.

b DALYs and costs discounted at a rate of 7%.

c Healthcare costs originally in PHP, converted to USD using 2020 average exchange rate (1 USD = 49.62 PHP); estimates of disease events in absolute counts.

d Vaccination program costs (discounted).

e Total program costs minus averted healthcare costs from government and societal perspective.

#### 3.1.1 Without RV vaccination

Over a 10-year period (2021–2031), total healthcare costs of RVGE without vaccination would be about $71 and $175 million USD from a government and a societal perspective, respectively. Concurrently, there would be an estimated 2 million cases of non-severe RVGE, 0.9 million cases of severe RVGE, and 65,000 cases of intussusception. In total, there would be about 1.3 million healthcare visits, 0.3 million hospitalizations, and 17,000 deaths due to RVGE.

#### 3.1.2 With RV vaccination

With an RV vaccination program in place, healthcare costs of RVGE would reduce by 42–44% over 10 years compared to no vaccination, with an estimated $30–31 million USD averted from a government perspective and about $72–76 million USD averted from a societal perspective. This would result in an incremental net cost of approximately $104, $105, $220, and $277 million USD, from a government perspective, for the inclusion of Rotavac, Rotasiil, Rotarix and Rotateq, respectively, in the NIP for a 10-year-period. From a societal perspective, there would be an incremental net cost of about $58, $60, $178, and $231 million USD for the inclusion of Rotavac, Rotasiil, Rotarix and Rotateq, respectively.

RV vaccination would also reduce cases of non-severe RVGE by 41–43% and severe RVGE by 48–51%, resulting in about 0.8 million non-severe RVGE and 0.4 million severe RVGE cases averted over 10 years. On the other hand, there would a potential increase of about 650 intussusception cases over 10 years, translating to an increase of 1% from baseline pre-vaccination cases. Overall, there would be a decrease in the number of hospital and clinic visits by approximately 0.5 to 0.6 million, hospitalizations by 0.15 million, and deaths by 8,000 over a 10-year period; these correspond to a reduction of 44–46%, 40–43%, and 35–37%, respectively.

### 3.2 Cost-effectiveness analyses

#### 3.2.1 Base case

In the base-case scenario, Rotavac was the least costly RV vaccine from both government and societal perspectives (Supplemental Fig. 1). The other three RV vaccines provided equivalent benefits but at higher costs. The cost-effectiveness of Rotavac (discounted US$ per DALY averted) was $1,148 ($830–$1,682) from a government perspective and $646 ($233–$1,277) from a societal perspective.

#### 3.2.2 One-way sensitivity analyses

Supplemental Figs. 2 and 3 show the sensitivity of results to changes in key parameters. To illustrate this, each vaccine is compared to no vaccination, and we estimate sensitivity in the average cost-effectiveness ratio (each vaccine compared to no vaccination). Rotavac and Rotasiil had ACERs less than the 1 times GDP threshold from both government and societal perspectives at 3%, 5%, and 10% discount rates. Furthermore, Rotavac and Rotasiil remained cost-effective in all scenarios using high and low estimates for all parameters, from both government and societal perspectives. Rotarix had ACERs greater than 1 times GDP threshold in scenarios with low disease event rates and low vaccine efficacy from a government perspective. To achieve equivalent ACERs to Rotavac/Rotasiil, the price per dose of Rotarix and Rotateq would need to be lower, around $2.50 and $1.12, respectively, representing a 65% and 80% reduction in price from the base case values.

#### 3.2.3 Probabilistic sensitivity analyses

**Fig. 1:**
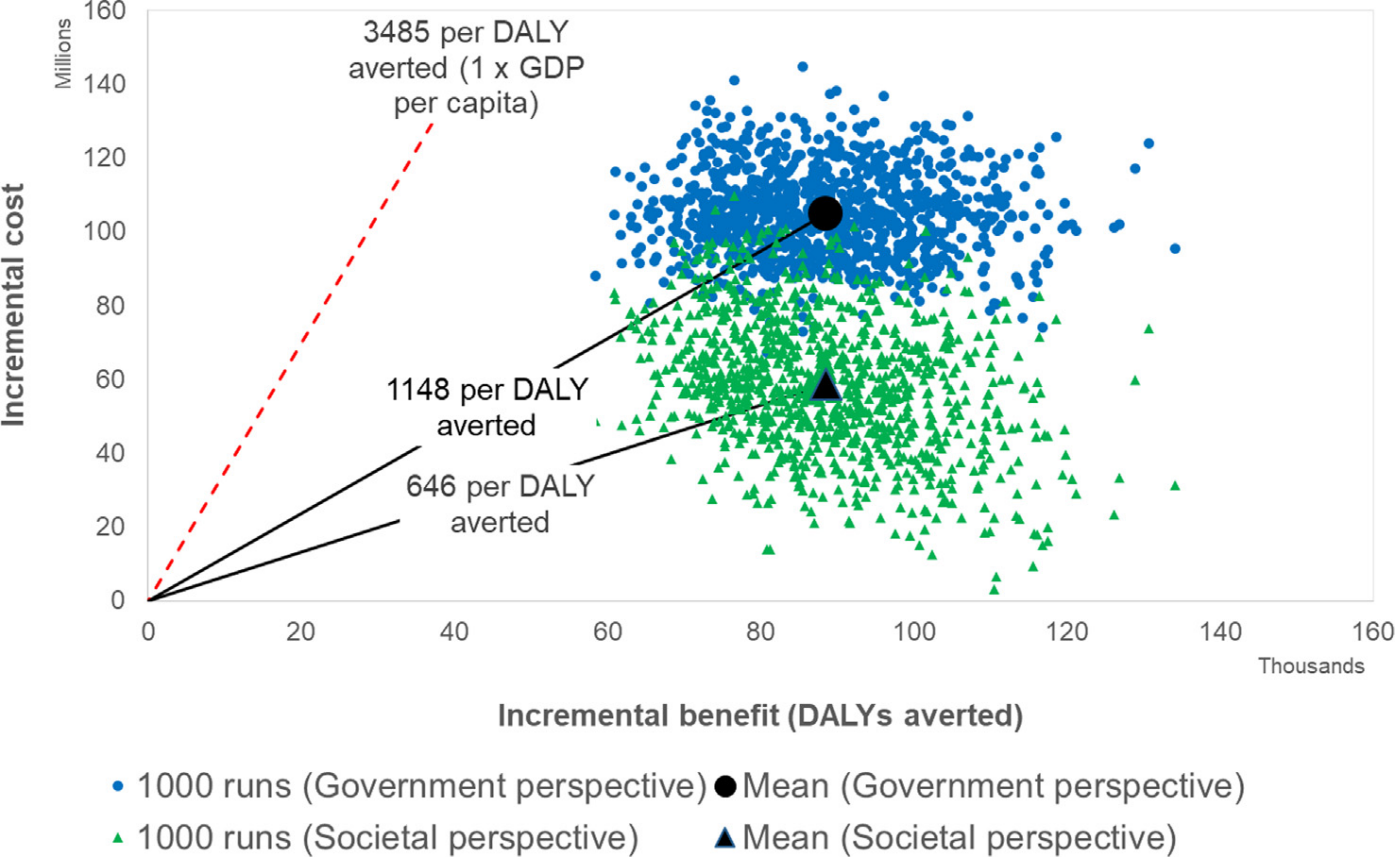
Cost-effectiveness of Rotavac compared to no vaccination (Government and societal perspectives).

**Fig. 2:**
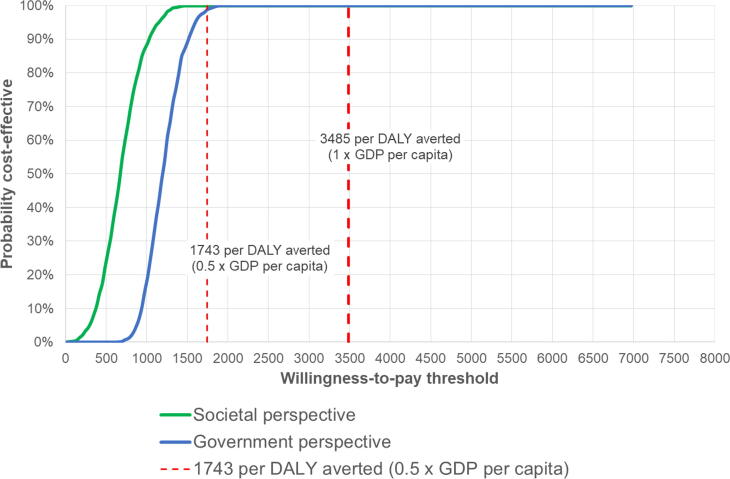
Probablity that Rotavac is cost-effective compared to no vaccination (government and sociatal perspectives).

**Fig. 3:**
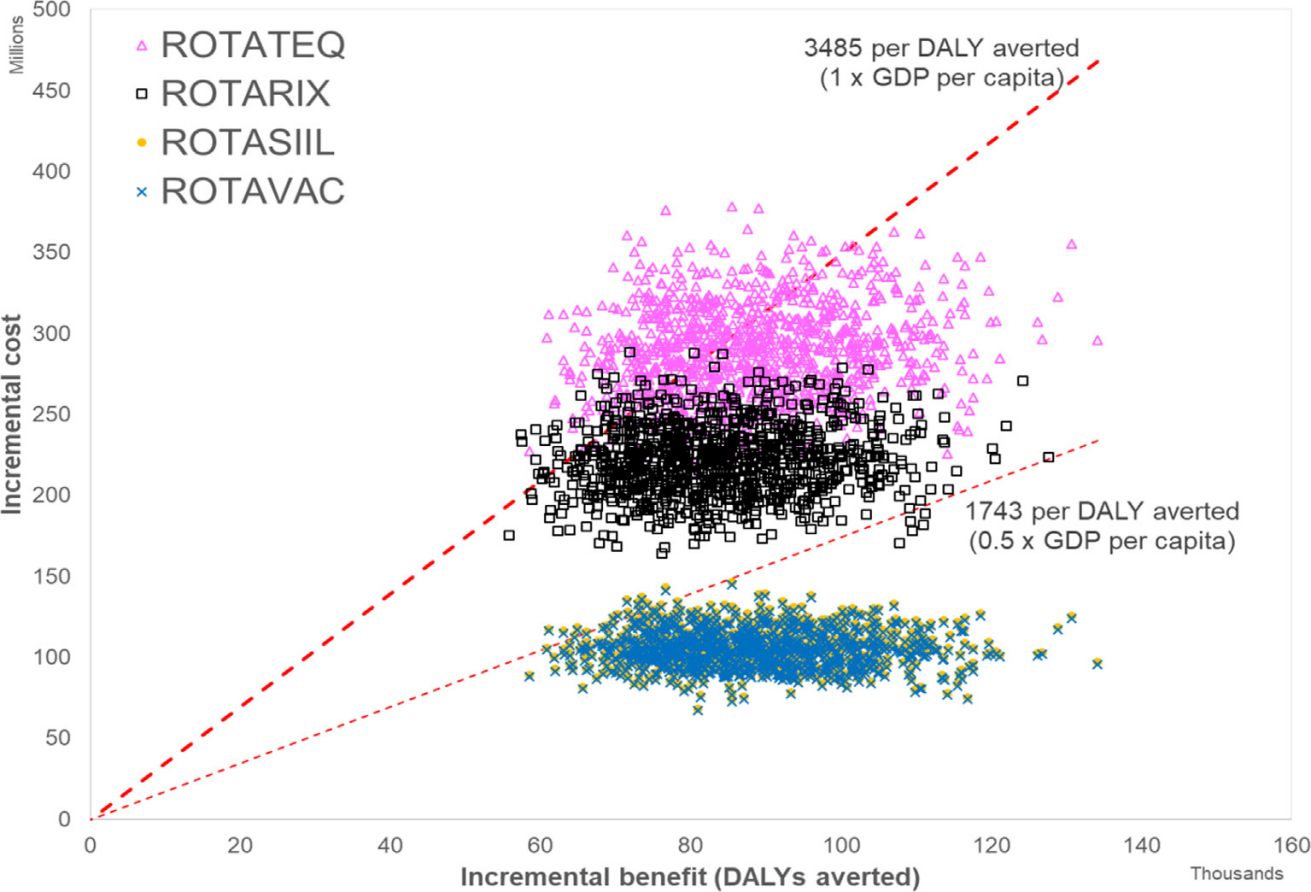
Cost-effectiveness plane showing costs and benefits of Rotavac, Rotasiil, Rotarix and Rotateq compared to no vaccination (government perspective). The plot above shows that both Rotavac and Rotasiil would be cost-effective when compared to no vaccination at a willingness to pay threshold set at 0.5 times the GDP per capita. While Rotavac has slightly lower costs, there is clearly considerable overlap between Rotavac and Rotasiil and it is not possible to say definitively which product is preferred. However, both products (Rotavac and Rotasiil) clearly dominate the other two products (Rotarix and Rotateq), because they provide the same benefit at a lower cost.

The least costly vaccine, Rotavac, has a 100% probability of being cost-effective, from both government and societal perspectives (Fig. 1, Fig. 2), at a willingness-to-pay threshold of 1 times GDP ($3,485). At a threshold of 0.5 times GDP ($1,743), it has a >99% probability of being cost-effective from a government and societal perspective. Fig. 3 shows that both Rotavac and Rotasiil would be cost-effective when compared to no vaccination at a willingness to pay threshold set at 0.5 times the GDP per capita. Thus, while Rotavac is marginally the least costly, there is clearly considerable overlap between Rotavac and Rotasiil, and it is not possible to say definitively which product is preferred. Fig. 3 also shows that both products (Rotavac and Rotasiil) clearly dominate the other two products (Rotarix and Rotateq) by providing the same benefit at a lower cost. If Rotavac was not available or licensed in the Philippines, then Rotasiil would be an attractive alternative, offering a similarly high probability of being cost-effective at 0.5 and 1 times the national GDP per capita. If neither Rotavac or Rotasill was available, then the alternative products (Rotarix and Rotateq) could be cost-effective using a threshold set at 1 times the national GDP per capita (95% and 71% probability to be cost-effective from a government perspective, respectively, and 100% and 93% probability to be cost-effective from a societal perspective, respectively). However, both products would need to be priced far more competitively to be considered cost-effective at a threshold of 0.5 times GDP per capita (1% and 0% chance to be cost-effective from a government perspective, respectively; and 18% and 2% chance to be cost-effective from a societal perspective, respectively).

## 4 Discussion

This timely and country-specific analysis revealed that among the four WHO-prequalified RV vaccines, Rotavac would be the least costly. Choosing either Rotasiil, Rotarix, or Rotateq would result in higher vaccine program costs with no additional health benefits. However, we should note that the vaccine prices we used in the model for Rotavac, Rotasiil, and Rotateq were estimates and may not be reflective of the actual future tender prices of the vaccines. In particular, the prices for Rotavac and Rotasiil were from the MI4A data from Gavi-eligible countries [42]. Procurement prices for these two RV vaccines from non-Gavi eligible countries were not available; hence, the actual tender prices may differ from those we used in this model, once these vaccines become available in the local market. This is important to note because the cost-effectiveness ratios from the base-case scenario and the sensitivity and probabilistic analyses show small differences in values between Rotavac and Rotasiil. In the PSA, both Rotavac and Rotasiil show similar high probabilities of being cost-effective using 0.5 and 1 times GDP per capita willingness-to-pay thresholds compared to no vaccination, and both products were assumed to be far less costly than Rotarix or Rotateq.

We assumed the same incremental health system costs for all the four vaccines, using the incremental health system cost of Rotarix as reference [44]. Rotarix does not require storage in freezers so the incremental health system costs we used as reference may be an underestimation for Rotavac, which requires storage in freezers to prolong its shelf life to five years [45]. Furthermore, in the Philippines there are island municipalities and areas geographically difficult to access, which means a vaccine requiring freezer storage conditions from a central area may incur additional substantial costs for transporting vaccines to vaccine-dispensing health centers. This would add to the incremental health system costs. On the other hand, Rotasiil, the next least costly RV vaccine, is stored in the same conditions as Rotarix and has been shown to be stable in ambient temperatures [45]. These factors may influence the choice of RV vaccine when these vaccines become available locally.

While the four vaccines similarly averted costs and disease events, the two-dose Rotarix resulted in slightly fewer averted costs and fewer averted events compared to the three-dose RV vaccines (Rotavac, Rotasiil, Rotateq). These differences are small, and the result of a third dose delivered a few weeks later and providing more durable overall protection for the three-dose vaccine products. However, as noted earlier, there are no known head-to-head studies demonstrating differential impact by product. If we assume all four have the same health impact, a Rotarix vaccination program over 10 years would still cost $116,580,596 USD more than a Rotavac vaccination program.

In our analysis, vaccine price is a crucial determinant of differential cost-effectiveness by vaccine type. To demonstrate, a Rotarix program with only two vaccine doses would incur less incremental health system costs than the three-dose vaccines; yet, due to a higher vaccine price per dose, the overall vaccination program cost for Rotarix was among the highest. The importance of vaccine price has been previously illustrated in a local CEA by Lam et al., who concluded that the 2015 prices of Rotarix and Rotateq warranted a reduction of about 70% in price for the vaccines to be cost-effective [16]. The vaccine price points we used in this study were 65% (for Rotarix) and 80% (for Rotateq) lower than those used in that study. Nevertheless, our two different CEAs have shown that vaccine price may change over time and affect future applicability of results. In fact, the potential entry of new vaccines into the local market may encourage more competitive pricing, possibly improving cost-effectiveness and budget impact of the precedents, Rotarix and Rotateq.

The cost-effectiveness ratios in our study can only be interpreted vis-à-vis our pre-defined willingness-to-pay threshold. While our set threshold has been used traditionally, there has been no explicit threshold in the Philippines from the payor’s side [22]. The significance of this threshold was exhibited in an RV vaccination CEA by Pempa et al. (2020) for Bhutan, where the threshold was fixed at only one-half of the country’s GDP per capita [41]. In their analysis, ICERs exceeded the threshold, and RV vaccination was not found cost-effective [41]. As seen on the cost-effectiveness acceptability curve from our PSA, even at 0.5 times GDP per capita threshold ($1,743), Rotavac and Rotasiil would still have a high probability of being cost-effective from both perspectives, while the other two vaccine types would not. These findings may further allude to the robust cost-effectiveness of Rotavac and Rotasiil. We also found Rotavac and Rotasiil would remain cost-effective at 0.5 times GPD capita thresholds even if prices for these products were to approximately double from the values used in this analysis. However, increasing prices might also reduce the affordability of these products.

In the Philippines, inclusion of vaccines in the NIP is also influenced by the ability to address health inequities [22]. In our analysis, healthcare costs were considerably higher from a societal perspective, despite nationwide coverage of RVGE hospitalization under a social health insurance scheme. These data may point to a large contribution from out-of-pocket costs and loss of household income. Such health expenditures for RVGE may result in medical impoverishment of economically marginalized households. This can be averted by RV vaccination among the poorest-quintile households in developing countries, as previously revealed by Chang et al. [46]. Collectively, this body of evidence may provide additional support to the inclusion and scale-up of RV vaccination in the Philippines NIP.

Notably, in our analysis, there could be a potential increase of intussusception cases over a 10-year period for the expansion to a national RV vaccination program. Should this potential risk be observed, we estimate that around 275 rotavirus hospitalizations could be prevented per intussusception hospitalization (or about 140 rotavirus deaths prevented per intussusception death). However, our estimates of the number of excess cases of intussusception were pessimistic for a number of reasons. First, we used pre-vaccine hospitalization rates from the WHO Western Pacific Region, which are far higher than other the rates in other WHO regions [32]. Second, we assumed that a substantial number of children would not have access to hospitals, and that a very high proportion (90%) of these untreated children would subsequently die, compared to 0.05% of children treated in hospitals. Third, we used estimates of the relative risk of intussusception among vaccine recipients based on a global pooled meta-analysis of self-controlled case series studies [21]. However, many of these studies are from high-income settings with demonstrated higher relative risks [47]. This may be related to varying risks associated with different populations [48]. Furthermore, because the studies included in this meta-analysis evaluated only Rotarix and Rotateq, the pooled risk ratio inputs used in the model may not apply to Rotavac and Rotasiil [21]. Indeed, neither vaccine has been associated with an elevated increased in intussusception to date.

While the WHO position strongly emphasizes that the benefits of RV vaccination on severe diarrhea far outweigh the risks for intussusception, it is recommended that active intussusception surveillance accompany the introduction of RV vaccination to monitor and assess this risk [48]. Presently, the Philippines does not have an active intussusception surveillance system in place. This surveillance will necessarily need additional financial resources, which will make vaccine program costs an even more important consideration in determining which RV vaccine will be the most affordable and cost-effective.

Our analysis included some limitations. First, we used international data for some model parameters, such as health system costs, thus restricting local applicability of our results. Although local disease rates for RVGE were available, the data originated from urbanized areas, which under-represented rural communities which may have a different disease burden. Furthermore, the UNIVAC model did not account for herd immunity, effects of breastfeeding, and possible long-term sequelae of RVGE such as malnutrition and adverse neurodevelopmental outcomes. Altogether these areas constitute current gaps in local research.

## 5 Conclusion

A rotavirus vaccination program over a 10-year period using any of the four WHO-prequalified RV vaccines (Rotavac, Rotasiil, Rotarix, and Rotateq) compared to no vaccination would avert 30–50% of visits, hospitalizations, and deaths from RVGE. Rotavac vaccination had the best value-for-money with the lowest ICER compared to no vaccination. However, given the uncertainty in actual vaccine prices, both Rotavac and Rotasiil are likely to be cost-effective options in the Philippines. Rotarix and Rotateq are expected to offer similar benefits but at a higher cost, so would need to be priced far more competitively for introduction consideration.

## Declaration of Competing Interest

The authors declare that they have no known competing financial interests or personal relationships that could have appeared to influence the work reported in this paper.
