# Supplementary material for: Cost-effectiveness of rotavirus vaccination in the Philippines: A modeling study
Source: Vaccine. 2021 Nov 26;39(48):7091–100. doi: 10.1016/j.vaccine.2021.09.075 (PMC8631456; doi:10.1016/j.vaccine.2021.09.075)

General Costing Methodology

After itemizing healthcare costs, we constructed point and interval estimates for each item, using different strategies detailed in later sections. The total cost of each item was its unit cost multiplied by the quantity then expressed as a range. The subtotals (in blue) corresponded to the costs from the government perspective while overall totals (in green) represented costs from the societal perspective. For visits in general, the low and high estimates were obtained first, then averaged to produce the mid-value. For hospitalizations, with the availability of PhilHealth data, we determined the point estimate before the interval estimate.

Costing Tables

Supplementary Table 1. Line item costs of non-severe RVGE visit.

| **Item** | **Quantity** | **Unit Cost (PHP)** | **Total Cost (PHP)** | **Ave. Cost (PHP)** |  |
| --- | --- | --- | --- | --- | --- |
| **Direct Medical Costs** |  |  |  |  |  |
| Physician visits | 2 | 250 - 500 | 500 - 1000 | 750 |  |
| Oral rehydration solution | 25 | 3.6 - 9 | 90 - 225 | 157.5 |  |
| Paracetamol | 1 | 16.5 - 45 | 16.5 - 45 | 30.75 |  |
| Zinc syrup | 3 | 41 - 100.75 | 123 - 302.25 | 212.625 |  |
| Routine stool test | 1 | 40 - 120 | 40 - 120 | 80 |  |
| Subtotal (PHP) | -- | -- | 769.5 - 1,692.25 | 1,230.88 |  |
| Subtotal (USD) | -- | -- | 15.51 - 34.10 | 24.81 |  |
| **Direct Non-medical Costs** |  |  |  |  |  |
| Diapers (5 cases/day x 5 days) | 25 | 5.4 - 6 | 135 - 150 | 142.5 |  |
| Transportation | 2 | 18 - 50 | 36 - 100 | 68 |  |
| **Indirect Costs** |  |  |  |  |  |
| Income loss | 1 | 459.11  (279.19 – 1,022.03) | 459.11  (279.19 – 1,022.03) | 459.11 |  |
| Meals | 1 | 52.09-70.18 | 52.09 - 70.18 | 61.14 |  |
| Overall total (PHP) | -- | -- | 1,271.78 - 3,058.46 | 1,961.63 |  |
| Overall total (USD) | -- | -- | 25.63 – 61.64 | 39.53 |  |

We assumed an episode of non-severe RVGE would entail two physician visits (initial and follow-up). For the low-end estimate, we used the hourly rate of a public primary care physician while for the high-end, we used the usual consultation fee of a pediatrician. Both estimates were based on expert opinion. The low-end unit costs of oral rehydration solution (200-ml sachet), paracetamol (250 mg/mL bottle), and zinc syrup (55 mg/5 mL bottle) were obtained from the DOH Drug Price Reference Index while the high-end costs came from a variety of pharmacies. The low and high cost estimates of a stool examination were taken from different DOH-retained hospitals. We also surveyed a number of pharmacies for the low and high unit costs of diapers. Meanwhile, quantities of the drugs and diapers were based on expert opinion. The unit cost of transportation accounted for travel to and from the physician’s clinic. Its low end was acquired from the current jeepney fare while the high end was the usual tricycle fare. We based the number of days of income loss on the CEA by Lee et al, but updated the cost estimates using 2017 lowest and highest daily basic salary (i.e., elementary occupations and managers, respectively).^14^ On the other hand, the costs of meals were derived from data of the Food and Nutrition Research Institute, with rural estimates serving as the lower end and urban estimates as the higher end.

Supplementary Table 2. Line item costs of severe RVGE visit.

| **Item** | **Quantity** | **Unit Cost (PHP)** | **Total Cost (PHP)** | **Ave. Cost (PHP)** |  |
| --- | --- | --- | --- | --- | --- |
| **Direct Medical Costs** |  |  |  |  |  |
| Intravenous fluids | 3 | 40.94 - 75 | 122.82 - 225 | 173.91 |  |
| Infusion set | 1 | 15 | 15 | 15 |  |
| Intravenous cannula | 1 | 15 | 15 | 15 |  |
| Alcohol swab | 1 | 75 - 85 | 75 - 85 | 80 |  |
| Surgical tape | 1 | 100 - 180 | 100 - 180 | 140 |  |
| Use of facility | 4 | 200 - 500 | 800 - 2,000 | 1600 |  |
| Non-severe RVGE visit | -- | -- | 769.50 - 1,692.25 | 1,230.88 |  |
| Subtotal (PHP) | -- | -- | 1,897.32 - 4,212.25 | 3,254.79 |  |
| Subtotal (USD) | -- | -- | 38.24 - 84.89 | 65.59 |  |
| **Direct Non-medical Costs** |  |  |  |  |  |
| Diapers (8 cases/day x 7 days) | 56 | 5.4 – 6.96 | 302.4 – 389.76 | 346.08 |  |
| Transportation | 2 | 150 - 200 | 300 - 400 | 350 |  |
| **Indirect Costs** |  |  |  |  |  |
| Income loss | 7 | 459.11  (279.19 – 1,022.03) | 3,213.77  (1,954.33 – 7,154.21) | 3,213.77 |  |
| Meals | 1 | 52.09 – 70.18 | 52.09 – 70.18 | 61.14 |  |
| Overall total (PHP) | -- | -- | 4,506.14 – 12,226.40 | 7,575.78 |  |
| Overall total (USD) | -- | -- | 90.81 – 246.40 | 152.68 |  |

We presumed emergency room (ER) visits for severe RVGE lasted 4 hours (see quantity listed for use of facility), based on the usual duration of hydration for moderate dehydration. During the ER stay, we assumed the child would warrant supplies for intravenous hydration, as enumerated in the table. The low and high costs of such medical equipment were canvassed across various pharmacies, although some items were found to have only standard price points. In contrast, the cost estimates for use of the facility were derived in a different manner. We first obtained the mid-value from a private hospital, then halved it for the low end as we increased it by 25% for the high end. After the ER stay, we assumed the child had not fully recovered and would need further medical attention, incurring costs equivalent to the direct medical costs of a non-severe RVGE visit. Costs of diapers were computed in a similar fashion to non-severe RVGE visits but with longer duration of illness. Transportation costs were inflated because households were usually farther from ERs than from clinics. Days of income loss were presumed longer than for non-severe RVGE because severe cases may require longer home care. Meals were computed as described previously.

Supplementary Table 3. Line item costs of severe RVGE hospitalization.

| **Item** | **Quantity** | **Unit Cost (PHP)** | **Total Cost (PHP)** | **Ave. Cost (PHP)** |  |
| --- | --- | --- | --- | --- | --- |
| **Direct Medical Costs** |  |  |  |  |  |
| Reimbursed cost | -- | 6,000.00  (4,500.00 – 7,500.00) | 6,000.00  (4,500.00 – 7,500.00) | 6,000.00 |  |
| Subtotal (PHP) | -- | -- | 6,000.00  (4,500.00 – 7,500.00) | 6,000.00 |  |
| Subtotal (USD) | -- | -- | 120.92  (90.69- 151.15) | 120.92 |  |
| Out-of-pocket costs | -- | 7,125.06  (894.94 – 20,855.18) | 7,125.06  (894.94 – 20,855.18) | 7,125.06 |  |
| Total cost of hospital bill | -- | 13,125.06  (5,394.94 -20,855.18) | 13,125.06  (5,394.94 -20,855.18) | 13,125.06 |  |
| **Direct Non-medical Costs** |  |  |  |  |  |
| Diapers (8 cases/day x 7 days) | 56 | 5.4 – 6.96 | 302.4 – 389.76 | 319.2 |  |
| Transportation | 2 | 150 - 200 | 300 - 400 | 350 |  |
| Antibiotics | -- | 13.75 – 125.00 | 13.75 – 125.00 | 65.5 |  |
| **Indirect Costs** |  |  |  |  |  |
| Income loss | 7 | 459.11  (279.19 – 1,022.03) | 3,213.77  (1,954.33 – 7,154.21) | 3,213.77 |  |
| Meals | 9 | 52.09 – 70.18 | 468.81 – 631.62 | 550.26 |  |
| Overall total (PHP) | -- | -- | 8,434.23 – 29,555.71 | 17,623.80 |  |
| Overall total (USD) | -- | -- | 169.98 – 595.64 | 355.18 |  |

Using PhilHealth data, we determined the mean of hospital bills filed for insurance claims under the ICD code A08.0 (rotaviral enteritis) from 2016 to 2018. We then derived the 95% confidence interval of the mean for our high and low cost estimates. The costs of the hospital bills were disaggregated into reimbursed costs (i.e., costs that were shouldered by the social health insurance company) and out-of-pocket costs (i.e., costs of the hospital bill that had exceeded the reimbursable amount and were shouldered by the household). The reimbursed amount is fixed every year, so we treated this as the base amount (mean) and used its +/- 25% as the low and high values. The average, low, and high cost estimates for out-of-pocket costs was the mean, with 95% confidence intervals. Only the PhilHealth-reimbursed costs were considered under a government perspective. Costs of diapers and transportation were calculated as severe RVGE visits. We added antibiotics to non-medical costs, as suggested by local experts, based on the observational study of Santos et al who found antibiotics were prescribed, albeit not rationally, to RVGE hospitalizations.^5^ The low and high unit costs of antibiotics represented the price of one bottle of metronidazole syrup from DPRI and surveyed pharmacies, respectively. We assumed a bottle would suffice for 4 days after discharge to complete a 7-day antibiotic course (inclusive of a 3-day hospital stay). Computation for income loss and transportation costs was similar to that in severe RVGE visits. On the other hand, we augmented the price of meals to account for 3 meals per day for 3 days in the hospital.

Supplementary Table 4. Line item costs of intussusception hospitalization.

| **Item** | **Quantity** | **Unit Cost (PHP)** | **Total Cost (PHP)** | **Ave. Cost (PHP)** |  |
| --- | --- | --- | --- | --- | --- |
| **Direct Medical Costs** |  |  |  |  |  |
| Reimbursed cost | -- | 10,100.00  (7,575.00 – 12,625.00) | 10,100.00  (7,575.00 – 12,625.00) | 10,100.00 |  |
| Subtotal (PHP) | -- | -- | 10,100.00  (7,575.00 – 12,625.00) | 10,100.00 |  |
| Subtotal (USD) | -- | -- | 203.55  (152.66 – 254.43) | 203.55 |  |
| Out-of-pocket costs | -- | 15,103.28  (7,857.67 – 22,348.89) | 15,103.28  (7,857.67 – 22,348.89) | 15,103.28 |  |
| Total cost of hospital bill | -- | 25,203.28  (15,432.67 – 34,973.89) | 25,203.28  (15,432.67 – 34,973.89) | 25,203.28 |  |
| **Direct Non-medical Costs** |  |  |  |  |  |
| Transportation | 2 | 150 - 200 | 300 - 400 | 350 |  |
| **Indirect Costs** |  |  |  |  |  |
| Income loss | 4.69 | 459.11  (279.19 – 1,022.03) | 2,153.23  (1,309.40 – 4,793.32) | 2,153.23 |  |
| Meals | 14.07 | 52.09 – 70.18 | 732.91 – 987.43 | 860.24 |  |
| Overall total (PHP) | -- | -- | 17,774.98 – 41,154.64 | 28,566.75 |  |
| Overall total (USD) | -- | -- | 358.22 – 829.40 | 575.71 |  |

All estimates of total cost of hospital bill, reimbursed cost, and out-of-pocket cost were produced the same way as severe RVGE hospitalization using PhilHealth data. Transportation costs were also similarly derived. Likewise, we computed for income loss and costs of meals as previously detailed, using the average duration of hospitalization for intussusception.

Supplementary Figure 1. Average cost-effectiveness ratios of rotavirus vaccines in the base-case scenario (RV vaccine compared to no vaccination)


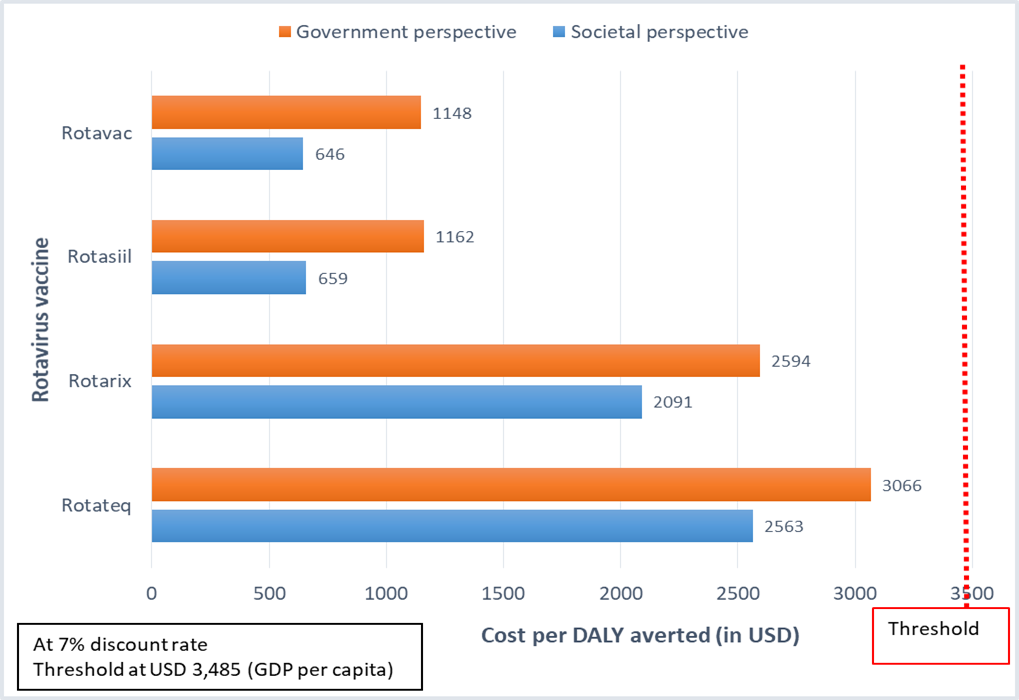


Supplementary Figure 2. One-way sensitivity analyses for Rotavac, Rotasiil, Rotarix and RotaTeq.


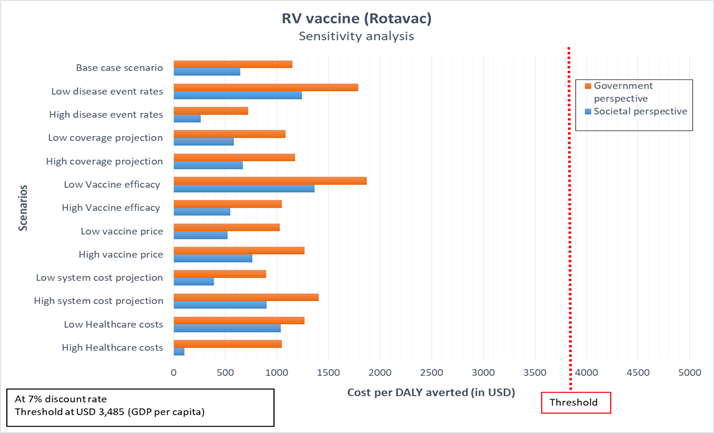

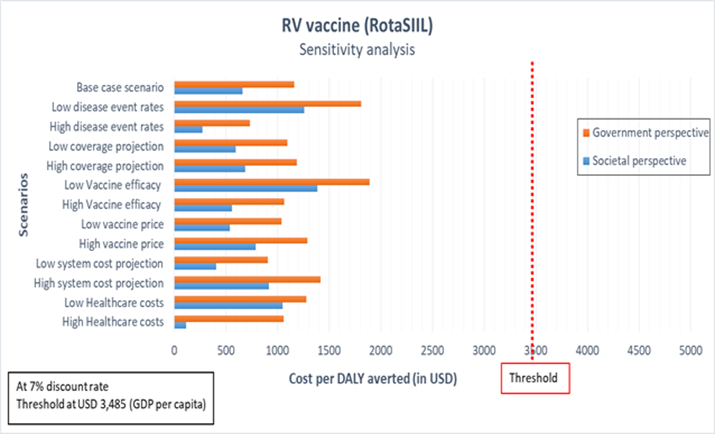


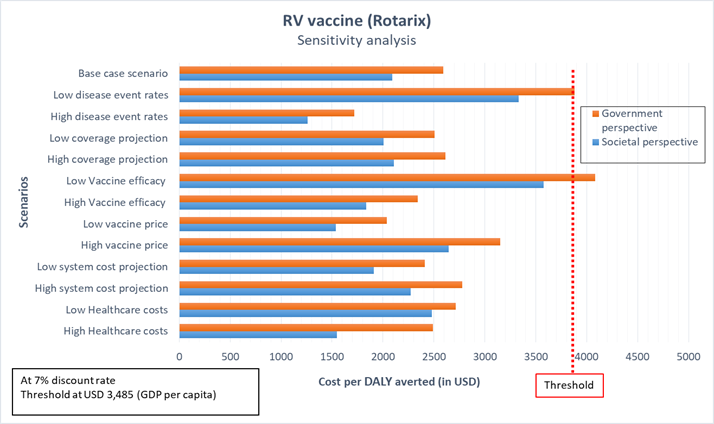

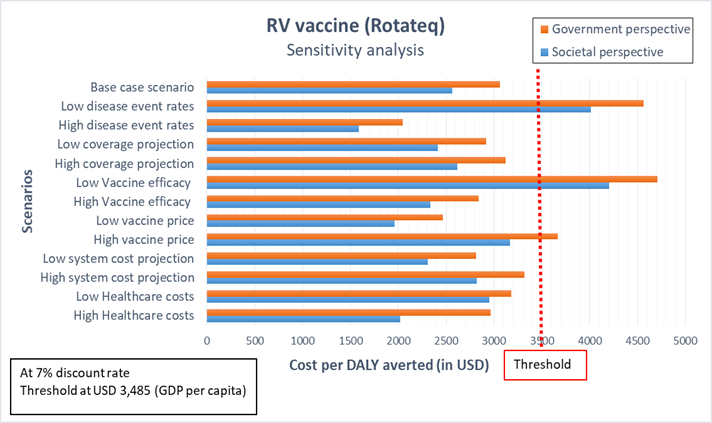


Supplementary Figure 3. Sensitivity analyses of discount rates for Rotarix, Rotavac, RotaSIIL, Rotarix, and RotaTeq


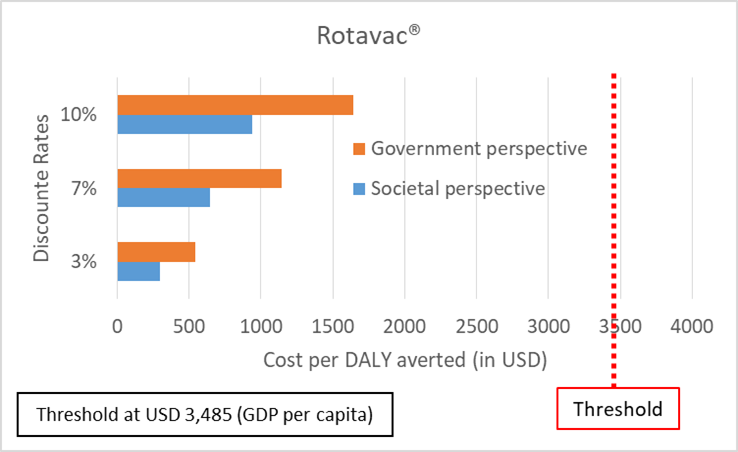

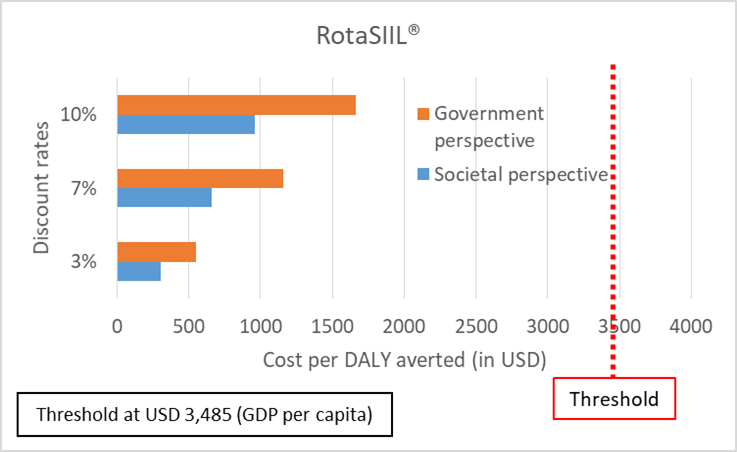


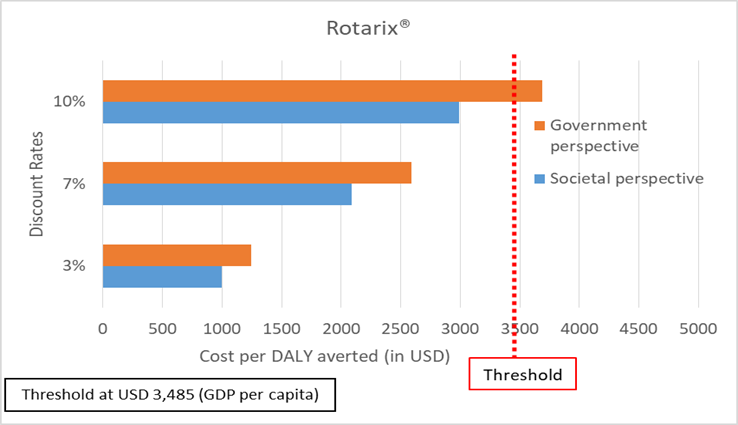

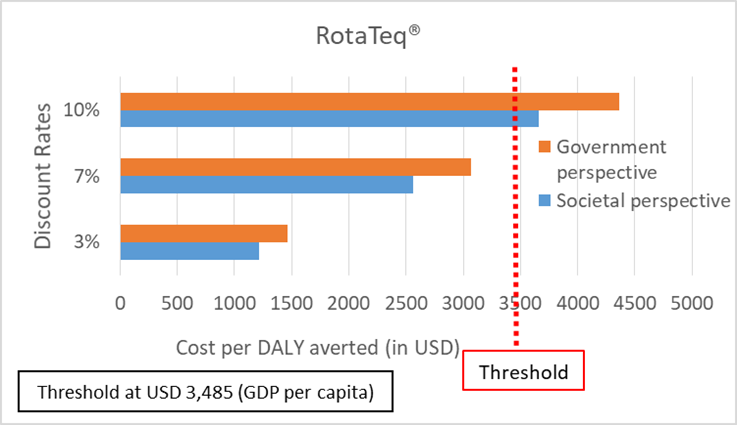

Supplement: Supplementary Data 1 [file mmc1.docx]
